# Great cormorants (*Phalacrocorax carbo*) as potential vectors for the dispersal of *Vibrio cholerae*

**DOI:** 10.1038/s41598-017-08434-8

**Published:** 2017-08-11

**Authors:** Sivan Laviad -Shitrit, Tidhar Lev-Ari, Gadi Katzir, Yehonatan Sharaby, Ido Izhaki, Malka Halpern

**Affiliations:** 1 Department of Evolutionary and Environmental Biology, Faculty of Natural Sciences, University of Haifa, Mount Carmel, Haifa Israel; 2 Department of Biology and Environment, Faculty of Natural Sciences, University of Haifa, Oranim, Tivon Israel

## Abstract

*Vibrio cholerae* is the cause of cholera, a devastating epidemic and pandemic disease. Despite its importance, the way of its global dissemination is unknown. *V*. *cholerae* is abundant in aquatic habitats and is known to be borne by copepods, chironomids and fishes. Our aim was to determine if fish-eating birds act as vectors in the spread of *V*. *cholerae* by consuming infected fish. We determined the existence of *V*. *cholerae* in the microbiome of 5/7 wild cormorants’ intestine. In three of these *V*. *cholerae*-positive wild cormorants, the presence of a gene for cholera toxin (*ctxA*) was detected. We subsequently tested eight captive, hand-reared cormorants, divided into two equal groups. Prior to the experiment, the feces of the cormorants were *V*. *cholerae*-negative. One group was fed exclusively on tilapias, which are naturally infected with *V*. *cholerae*, and the other was fed exclusively on goldfish or on koi that were *V*. *cholerae*-negative. We detected *V*. *cholerae* in the feces of the tilapia-fed, but not in the goldfish/koi-fed, cormorants. Hence, we demonstrate that fish-eating birds can be infected with *V*. *cholerae* from their fish prey. The large-scale movements of many fish-eating birds provide a potential mechanism for the global distribution of *V*. *cholerae*.

## Introduction

*Vibrio cholerae* is the etiologic agent of cholera, a devastating diarrheal disease which causes epidemics and pandemics. The bacteria are endemic in the aquatic environments^1^. Copepods and chironomids are both considered natural reservoirs of *V*. *cholerae* ^1–5^. However, it is still not clear how this bacterium spreads all over the world^6^.

Green and Sanches^7^ and Frisch *et al*.^8^ found that chironomids and copepods can be transferred across waterbodies via waterbirds. Considering these findings we hypothesized that *V*. *cholerae* may be dispersed by migratory waterbirds, which consume chironomids or copepods (endozoochory) or carry them externally (epizoochory)^6, 9^. Support for this hypothesis was also found in the literature^10, 11^. Furthermore, many fish species feed on copepods and chironomids^12^, hence may act as a vector of cholera. Indeed, we recently demonstrated that fishes are also reservoirs of *V*. *cholerae* ^13^. This finding was supported by evidence from the literature that cholera cases have been associated with eating, consumption and cleaning of different fish species in different parts of the world^14–18^.

Because fish are commonly consumed by various waterbird species, they may also create a link between *V*. *cholerae* and waterbirds^6, 9^. Buck^19^ studied the presence of halophilic *Vibrios* and *Candida albicans* from different bird species in the USA and found these pathogens in the feces of one double-crested cormorant (*Phalacrocorax carbo*). *Campylobacter*, *Escherichia coli* and *Salmonella* were cultured from cloacal and pharyngeal swabs of double-crested cormorant chicks in Canada^20^.

Great cormorants are known as generalist foragers^21^ or specialist piscivores^22^, with a variety of regional^23^ and seasonal^24^ diets. They are opportunistic predators that consume a wide range of fish species of diverse size^25, 26^. Recently, cormorants have increased in number of individuals, thereby causing trouble for commercial and sports fisheries in lakes and rivers all over Eurasia^27, 28^ and North America (*Phalacrocorax auratus*)^29^.

A rise in the numbers of great cormorants arriving from Europe to over-winter in Israel from October to March has been demonstrated. The figures are between 17,000 and 29,000 individuals^30^. This large number of fish-eating birds causes problems for the fish industry in Israel, as has been described for other countries. As a result, the Israeli authorities allow each fish farm to shoot down up to six cormorants per fish farm per day^30^.

Here we studied the microbiome composition of the cormorant’s intestine and the possible role of fish infected with *V*. *cholerae* in transferring the bacteria to the birds. Our current results reveal that different areas of the cormorant intestine have a qualitatively unique bacterial composition, even though the microbiome of each individual differs from the microbiomes of all the others. Additionally, we have demonstrated that by consuming fish naturally infected with *V*. *cholerae*, great cormorants get infected with this bacterial species. This infection may last up to 72 h. Our study opens an exciting new direction for understanding the global spread of *V*. *cholerae*.

## Results

### Analyses of wild great cormorants’ intestine microbiome

**Figure 1:**
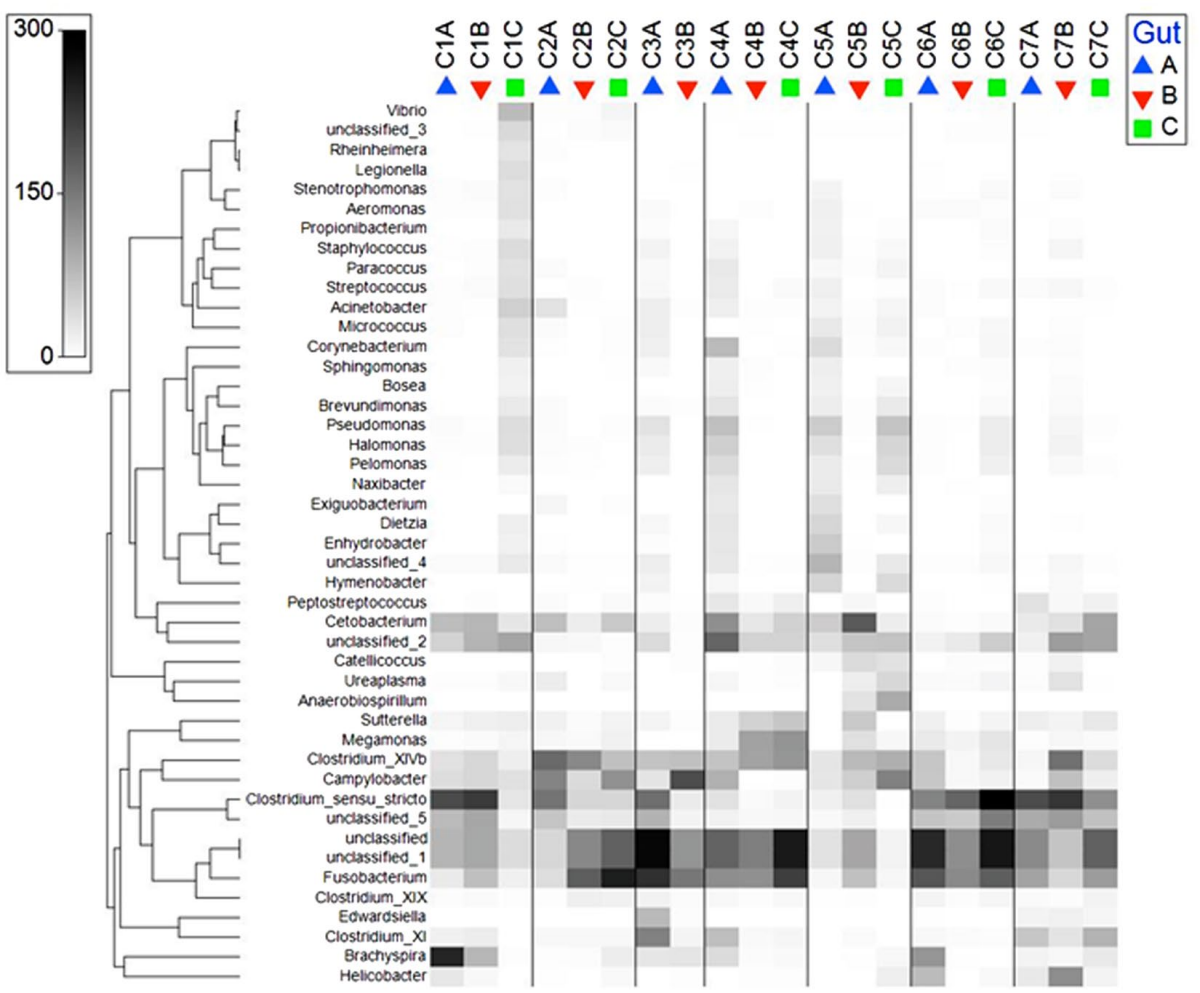
A shade plot presentation of data matrix of all samples showing the dominant OTUs (with minimum of 500 sequences per each OTU in all the samples together) and their taxonomic classification. The different samples are presented in columns. The numbers in the sample name indicate the cormorant individuals; the letters (at the end of the name) A, B, C indicate the three intestine parts: A - esophagus, B - middle, C - cloaca. The grey scale squares are in linear proportion to a square root transformation of the relative abundance of each presented genus or unclassified OTU in a sample. White squares signify the absence of the genera or OTU in a sample. Clustering of the genera (y-axis) was due to similar distribution across the samples. The figure was drawn with the primer 7 software (http://www.primer-e.com).

Bacterial communities of intestine samples from the seven great cormorants were analyzed by sequencing the V4 variable region of the bacterial 16S ribosomal RNA (rRNA) gene on an Illumina MiSeq platform. Three intestine areas were examined for each bird (total of 20 samples). 1,245,131 quality sequences were obtained. These were classified into 59,299 operational taxonomic units (OTUs) using the cutoff of 97% sequence similarities. Subsampling according to the smallest sample (24,901 sequences) resulted in 498,020 sequences, which were classified into 33,315 OTUs. The dominant OTUs with minimum of 500 sequences in all the samples in total and their taxonomic classification are shown in Fig. 1.

Rarefaction curves, which describe the OTU numbers as a function of the sampling effort (total sequences), were performed at a phylogenetic distance of 3% sequence similarity for all intestine samples (Fig. S1). Although all samples had at least 40,000 sequences, the rarefaction curve of none of the samples reached an asymptote level, demonstrating that our sampling effort was not sufficient to obtain an accurate estimate of OTU richness.

**Figure 2:**
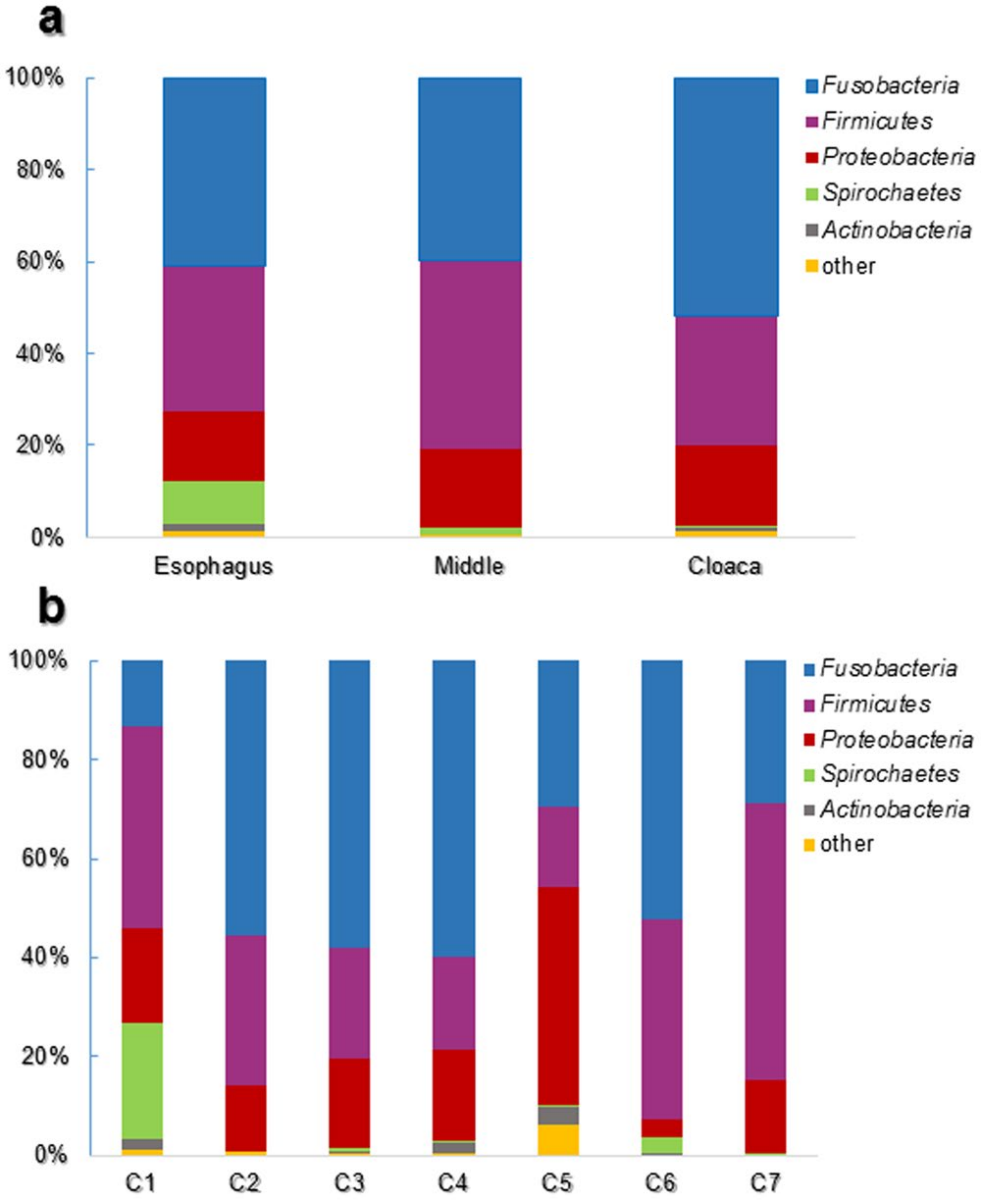
Average OTU abundances at the phyla level. (**a**) Sum of all the samples from a specific section (esophagus, middle, cloaca) of the cormorant intestine. *Fusobacteria*, *Firmicutes Actinobacteria* and *Proteobacteria* showed no significant differences in the different intestine parts (p > 0.05). However, *Spirochaetes* phylum evinced significant differences in relative abundance in the different intestine parts (repeated measures ANOVA: F_2,10_ = 4.84, p = 0.034). (**b**) Sum of the phyla abundances from the three sampled areas (esophagus, middle, cloaca) for each individual cormorant. *Fusobacteria* was the most abundant phylum in birds C2, C3, C4 and C6, *Firmicutes*, the most abundant phylum in birds C1 and C7 and *Proteobacteria*, the most abundant phylum in bird C5.

Overall, 13 phyla were detected. *Fusobacteria*, *Firmicutes* and *Proteobacteria* were the dominant phyla in all samples (Fig. 2a and b). The phylum *Fusobacteria* was the most abundant in samples that belonged to the esophagus and the cloacal regions along the intestine, the vast majority of reads belonged to the genus *Fusobacterium* (Fig. 1). *Firmicutes* was the most abundant phylum in the middle part of the intestine and the majority of reads belonged to the genus *Clostridium* (Fig. 1). The prevalence of *Proteobacteria* was 17–19% in all the intestine parts, with *Campylobacter* as the relatively dominant genus (4.6%) (Fig. 1). *Spirochaetes* phylum was found in all intestine sections, at relatively lower abundances (esophagus, 9.1%; middle, 1.24%; cloaca, 0.1%). The dominant genus in this phylum was *Brachyspira*. *Actinobacteria* was present in the esophagus and the cloaca regions (1.8% and 1%, respectively) with more than one dominant genus (*Corynebacterium*, *Dietzia* and *Micrococcus*) (Fig. 1). Significant differences were observed between the relative abundances of *Spirochaetes* phylum in the three different parts along the cormorant’s digestive tract (Repeated measures ANOVA: F_2,10_ = 4.84, p = 0.034). No significant differences were detected among *Fusobacteria*, *Firmicutes*, *Actinobacteria* and *Proteobacteria* in the different intestine parts in the cormorants’ digestive tracts (p > 0.05) (Figs 1 and 2a).

Differences among all individuals at the phylum level (Fig. 2b) revealed that *Fusobacteria* was the most dominant phylum in four out of the seven examined birds. In birds C1 and C7 the dominant phylum was *Firmicutes* (40.8% and 55.5%, respectively); however, in cormorant C5 the dominant phylum was *Proteobacteria* (44.1%). 23.6% and 3.2% of the OTUs belonged to phylum *Spirochaetes* in cormorants C1 and C6, respectively; in all the other birds this phylum was present in less than 1% of the OTUs (Fig. 2b).

According to the Sobs mean (observed number of species in a sample) and Chao1 (species diversity) estimators, OTU richness was the same in the three different intestine sections; the expected bacterial OTU richness was also similar (Table 1). In sum, the different sampled intestine parts were alike in their observed and expected bacterial OTUs. By contrast, when Sobs mean and Chao1 estimators were calculated for the three pooled sections from each bird, OTU richness of cormorant C1 was less than that of the others, and the richness of cormorant C7 was much higher than that of the others (178 ± 54 and 377 ± 15, respectively) (Table S1).

**Table 1:** Microbial richness of the three intestine parts in all cormorants (subsampled OTUs at the genera levels).

Phylogenetic level	Intestine part	A	B	C
OTUs	Sobs Mean ± SD	203 ± 85.0	229 ± 111.0	267 ± 131.0
	Chao1 ± SD	1138 ± 777.0	1221 ± 709.0	1387 ± 917.0
All genera	Sobs Mean ± SD	202 ± 7.0	191 ± 7.0	229 ± 8.0
	Chao1 ± SD	217 ± 8.7	213 ± 15.6	249 ± 10.7

The indexes Sobs Mean and Chao1 were calculated on the EstimateS (Version 9.1.0) software. Sobs Mean was calculated as the average number of all taxonomic units in all samples and Chao1 was calculated as the expected taxonomic richness for the complete collection of each intestine part (more details can be found in the Methods section). A - esophagus, B - middle, C - cloaca.

**Figure 3:**
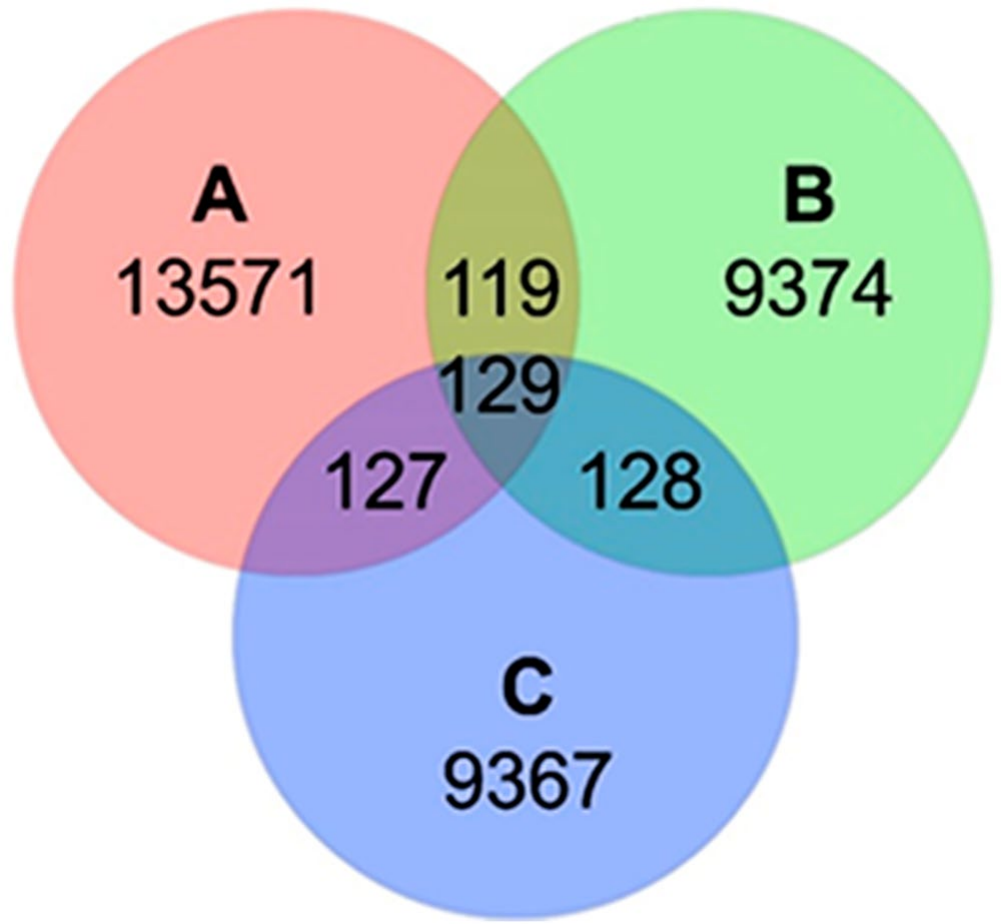
Venn Diagram at a distance of 0.03, illustrating the number of unique and shared subsampled OTUs in the libraries of bacterial communities of the three different cormorant intestine sections (A, B, C). A - esophagus, B - middle, C - cloaca.

**Figure 4:**
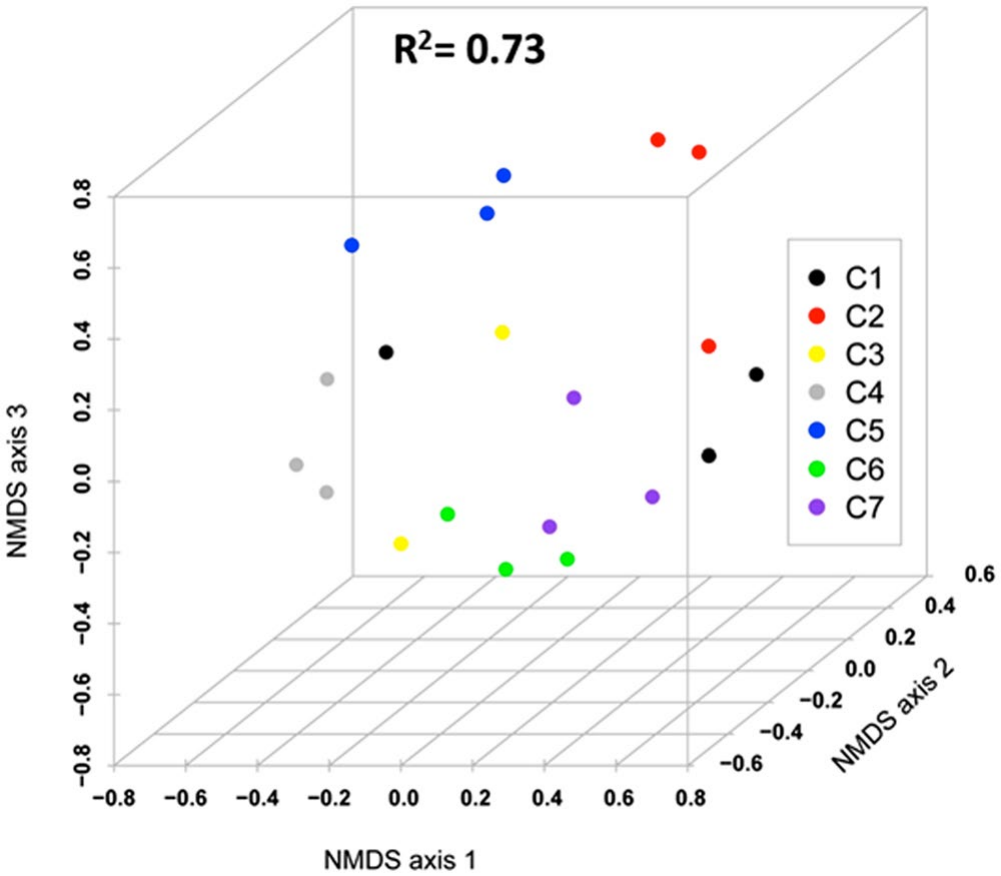
nMDS (nonmetric multidimensional scaling) plot of the entire bacterial community in each intestine sample. OTUs were determined based on 97% read similarities. Samples from the different intestine sections (esophagus, middle, cloaca) of each bird, clustered together. C1–C7 indicate the different cormorant individuals and are marked in different colors (three samples from an individual cormorant) (R^2^ = 0.73, stress value = 0.17). The different cormorant individuals were found to differ significantly in the intestine microbiome (ANOSIM; n = 7, R = 0.505, p < 0.01).

Venn diagram (Fig. 3) presents the shared and unique OTUs in each intestine section where all seven cormorants were pooled. The esophagus section contained the largest portion of unique OTUs, namely 40.7% of them. The middle and the cloaca sections had similar portions (29.6% and 28.1%, respectively). Only 129 OTUs (0.38%) overlapped all three intestine parts, indicating that each intestine section hosted a unique bacterial community. Venn diagram of all the three sections of each bird alone showed a similar profile (data not shown). The Venn diagram does not take into account differences in OTUs’ relative abundances. So when a quantitative approach was taken, using a three-dimensional non-metric multidimensional scaling (nMDS) analysis, the samples of each intestine part in a specific bird clustered together, suggesting that each bird hosts a unique bacterial community (R^2^ = 0.73, stress value = 0.17) (Fig. 4). Analysis of similarity (ANOSIM) verified the significant differences in the quantitative bacterial communities’ composition in the different cormorant individuals (n = 7, R = 0.505, p < 0.01). Thus, each bird hosts a significantly different microbiome composition and these differences distort and blur the differences in microbiome composition in different “areas” of the gut.

### Culturable and uncultured *Vibrios* and other potential pathogens

**Figure 5:**
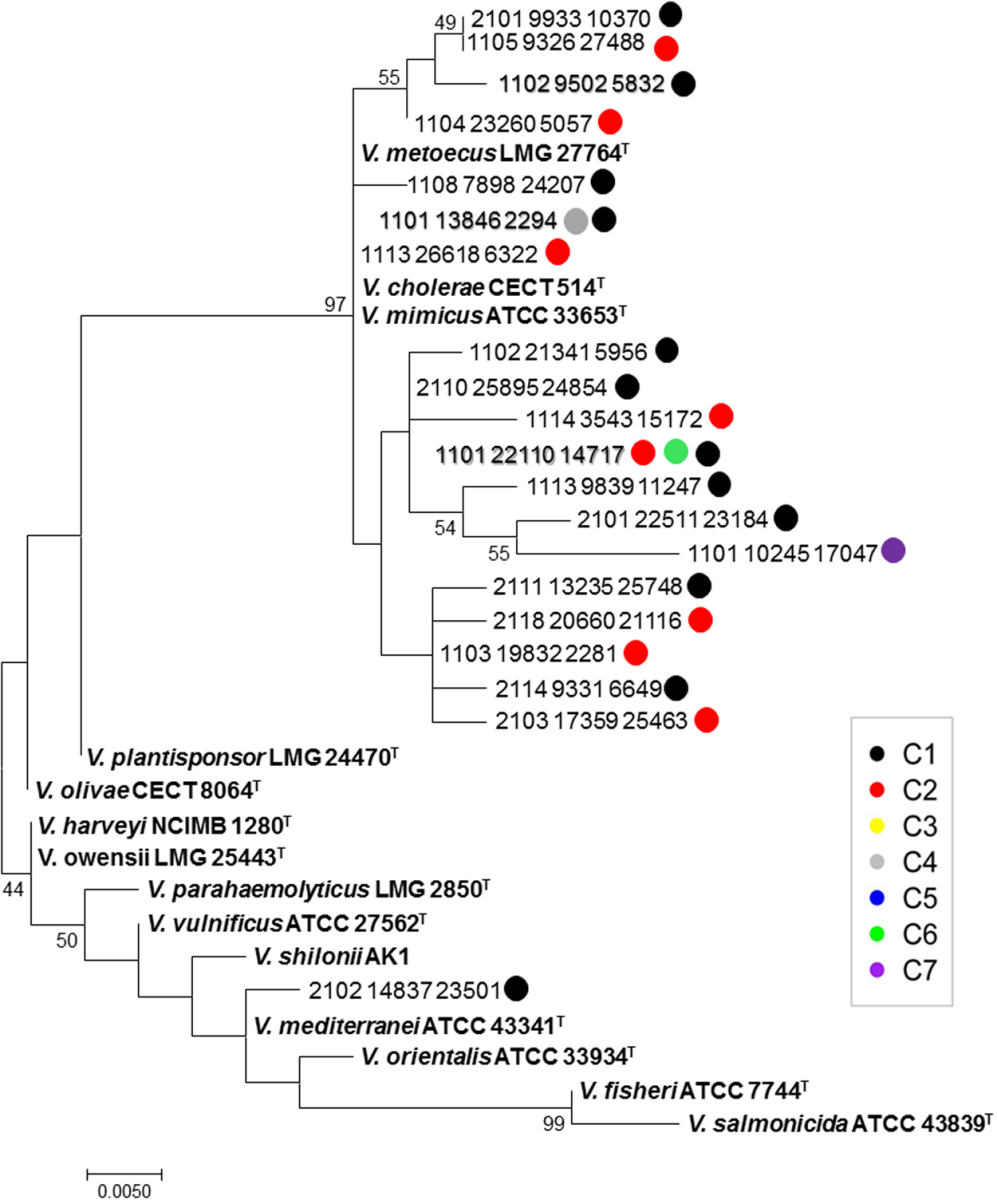
A maximum likelihood tree (generated in MEGA 5.0 software), based on the 16S rRNA gene sequences, showing the nearest neighbors of the most abundant *Vibrio* OTUs’ sequences. The different cormorant individuals (C1–C7) are marked in different colors and indicate the sequence origin from the different cormorant individuals. Bootstrap values (>50%) resulting from 1,000 replicates are indicated as percentages at branching nodes. Bar, 0.005 substitutions per nucleotide position. No *Vibrio* OTUs were detected in cormorants 3 and 5. The three most dominant OTUs (1101_22110_14717; 1102_9502_5832 and 1101_13846_2294) were marked in bold. These OTUs comprised of 1,845, 944 and 346 sequences, respectively.

*V*. *cholerae* was successfully cultured from only one of the seven wild cormorants’ intestine samples. However, the genus *Vibrio* was detected in five of the seven birds when an Illumina MiSeq sequencing platform was used. In total, 55 OTUs were identified as *Vibrios*. To ascertain the OTUs’ phylogenetic position, a phylogenetic tree was generated with representatives of the dominant *Vibrio* OTUs’ sequences. The tree revealed that all but one of the OTUs clustered together with *V*. *cholerae*, *V*. *mimicus* and *V*. *metoecus* cluster; the exception clustered with *V*. *mediterranei* (Fig. 5). Interestingly, 99.8% of the *Vibrio* OTUs were identified in the cloacal region.

To verify the presence of *V*. *cholerae* in the wild cormorant intestine samples, we looked for the presence of two *V*. *cholerae*-specific genes (*ompW* and *ctxA*) in the intestine DNA extractions. *ompW*, a specific outer membrane protein gene of *V*. *cholerae* was detected in cormorants C1, C2, C4, C6 and C7. These are the same birds in which *V*. *cholerae* OTUs were detected (Fig. 5). An interesting result was the detection of *ctxA* gene (encoding the A subunit of cholera toxin) in wild cormorants C1, C4, and C7 demonstrating that these birds harbored pathogenic strain of *V*. *cholerae*.

Among the observed OTUs, genera with potential pathogenicity for humans and/or birds, such as *Corynebacterium*, *Mycobacterium*, *Campylobacter*, *Helicobacter*, *Yersinia*, *Haemophilus*, *Clostridium* and more, were also detected (Table S2). Overall, genera related to human or bird pathogenic species, were found within the microbial community at a prevalence of 22.1% and 5.8%, respectively in all birds.

### Cormorants’ feeding experiments

To demonstrate that fish infected with *V*. *cholerae* transfer the bacteria to cormorants, we used cormorants (n = 8) hand-reared in captivity. Prior to the feeding experiments, the cormorants were fed on goldfish (*Carassius auratus*) or koi (*Cyprinos carpio*). Culturable *V*. *cholerae* was not detected in the intestines of goldfish (n = 10) or koi (n = 10). The same results were obtained (prior to the feeding experiment) for the feces of cormorants that were fed on these fish species. By contrast, the intestine of fresh collected tilapia (*Oreochromis niloticus* X *Oreochromis aureus*; n = 10) was found to be *V*. *cholerae*-positive.

**Figure 6:**
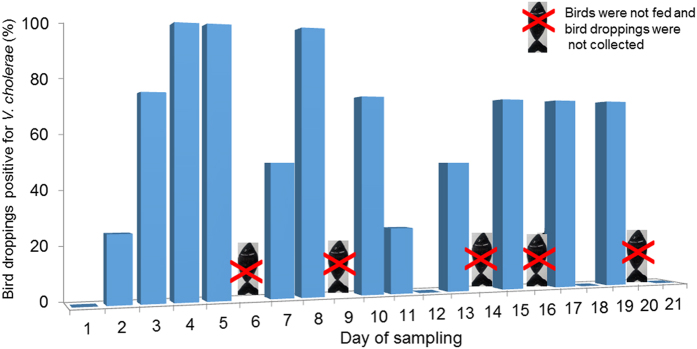
Hand-reared cormorants feeding experiment. Detailed results for treatment A (birds fed on tilapia fish). On day 1, all the bird droppings were negative for *V*. *cholerae*. A fish marked X represents days when the birds were not fed and droppings were not collected (n = 4).

During the experiment, the cormorants were divided into two groups (A, B) of four birds each. In the first experiment, group A was fed exclusively on tilapia and group B was fed exclusively on goldfish or koi. This procedure lasted for three weeks, after which the diet of group A was switched from tilapia to goldfish or koi for at least two weeks more (interval between experiments). Accordingly, in the interval between the experiments all cormorants from both groups A and B were fed on goldfish or koi. In the second experiment, diets were switched between the groups so that group A was fed on goldfish or koi while group B was fed on tilapia. Diet switching between the groups including the intervals between experiments was repeated seven times and included switching the diets between groups A and B. Four experimental repetitions are demonstrated in Figs 6 and S2. The figures show the success in isolating *V*. *cholerae* from the birds’ feces that were fed on tilapia. In all cases the feces of the cormorants fed on goldfish and koi were *V*. *cholerae*-negative while most of the feces of the cormorants fed on tilapia were *V*. *cholerae*-positive. In some experimental repetitions *V*. *cholerae* was isolated from birds’ feces within less than two hours of their feeding on tilapia (Fig. S2a–c).

Additionally, during the interval, while the birds’ diet was switched from tilapia to goldfish or koi, their droppings were *V*. *cholerae* positive even 72 h after their diet was switched from tilapia to goldfish or koi (experiment repetition no. 6). In experiment repetitions nos. 1 and 7, *V*. *cholerae* was detected 48 h after the diet switch, and in experiment repetitions nos. 2 and 4, *V*. *cholerae* was detected 24 h after the diet switch (Table 2). In marked contrast, *V*. *cholerae* was not isolated from the feces of the birds in the group fed exclusively on goldfish or koi. Hence, these results clearly demonstrate that *V*. *cholerae* can survive in cormorants’ digestive tract at least 72 h after ingestion of *V*. *cholerae*-infected fish (Table 2).

**Table 2:** The persistence of *V*. *cholerae* in the cormorants’ feces, days after their diets were switched from tilapia to golden fish or koi.

Feeding experiment	Time after switching the diet from tilapia to goldfish or koi		
	24 h	48 h	72 h
1	+	+	−
2	+	−	−
3	−	−	−
4	+	−	−
5	−	−	−
6	+	+	+
7	+	+	−

The results demonstrate that *V*. *cholerae* can survive in the cormorants’ digestive tract up to 72 h after ingestion of tilapia (fish colonized by *V*. *cholerae*).

### Virulence genes in *V. cholerae* isolates

Overall, 48 and 141 *V*. *cholerae* strains were isolated from fish and cormorants, respectively. When the presence of virulence genes was screened in the *V*. *cholerae* isolates, all isolates from the birds’ feces and the fish intestine were found positive for *ompW* and *toxR* genes and negative for *ctxA*, *tcpA* and *tcpI*. In the birds’ feces isolates, 14 different virulence genotypes were observed (Table 3). All isolates were positive for *hapA* gene. Most strains were positive for *hylA* gene (98%), 39% were *ompU*-positive, 26% possessed all examined TTSS genes and only 3.5% demonstrated the presence of *zot* gene (Table 3). Among the fish isolates, 13 different virulence genotypes were observed. Most isolates were *hylA*- and *hapA*-positive (96% and 83%, respectively), about 40% were *ompU*-positive and possessed all examined TTSS genes, and 23% were *zot*-positive (Table 3).

**Table 3:** Virulence traits of *V*. *cholerae* isolates from the cormorants’ droppings (n = 141) and fish intestines (n = 48).

No. of strains	Genotype prevalence%	Presence or absence of virulent genes*				
		TTSS^*^	*ompU*	*hlyA*	*zot*	*hapA*
**Isolates origin**: **cormorants’ feces**						
1	0.7	+	+	+	+	+
9	6.4	+	+	+	−	+
1	0.7	+	−	+	+	+
24	17.0	+	−	+	−	+
1	0.7	+	−	−	−	+
1	0.7	+	+	−	−	+
7	5.0	+/−	+	+	−	+
1	0.7	+/−	−	+	+	+
23	16.3	+/−	−	+	−	+
1	0.7	+/−	−	−	−	+
36	25.5	−	+	+	−	+
1	0.7	−	−	+	+	+
34	24.1	−	−	+	−	+
1	0.7	−	+	+	+	+
**Isolates origin**: **fish intestine**						
4	8.3	+	−	+	−	+
3	6.3	+/−	+	+	−	+
1	2.1	+/−	−	+	+	+
10	20.8	+/−	−	+	−	+
2	4.2	+/−	−	+	−	−
1	2.1	+/−	−	−	+	+
5	10.4	−	+	+	+	+
5	10.4	−	+	+	−	+
4	8.3	−	−	+	+	+
6	12.5	−	−	+	−	+
3	6.3	−	+	+	−	−
3	6.3	−	−	+	−	−
1	2.1	−	+	−	−	+

All the examined isolates were positive for *toxR* gene and negative for *ctxA*, *tcpA* and *tcpI* genes.

*PCR-based detection of the TTSS cluster (genes; *vcsC2*, *vcsN2*, *vspD*, and *vcsV2*). (+) positive, (−) negative, (+/−) positive for some but not all the genes in the TTSS cluster.

## Discussion

*V*. *cholerae* is the cause of the devastating epidemic and pandemic cholera disease. Massive cholera outbreaks are caused by the particular serogroups O1 and O139 which produce the cholera enterotoxin. An example is the case of the Haiti cholera outbreak following the 2010 earthquake^31, 32^. It is estimated that from 2008 to 2012 ca. 2.9 million people were infected yearly, with a death toll of ca. 95,000 worldwide^33^. *V*. *cholerae* is part of the natural flora and ecology of surface water^34^, where the bacteria commonly associate with zooplankton, particularly copepods^2^ and chironomids^3–5^, which are their potential vectors. It is estimated that both the pathogenic and non-pathogenic serogroups share the same niches^34^. Despite intensive efforts, the mechanisms that enable the widespread and rapid dispersion of *V*. *cholerae* remain an enigma. Some evidence supports our hypothesis^6^ that fish^13^ and waterbirds may act as intermediate reservoirs and vectors of *V*. *cholerae* strains, including pathogenic *V*. *cholerae* O1/O139 serogroups^35^. Particularly, Hounmanou *et al*.^36^ isolated *V*. *cholerae* O1 *ctxA* positive from tilapia. Moreover, *V*. *cholerae* was isolated from more than 20 species of waterbirds with pandemic O1 serogroups identified in great blue herons and ring-billed gulls^10, 11^.

In agreement with this hypothesis, we have demonstrated here that a most common fish-eating bird, the great cormorant, shed *V*. *cholerae* within a few hours of consuming fish which are colonized by *V*. *cholerae* (Fig. 6). *V*. *cholerae* colonization in the birds’ intestine may persist for at least 72 h (Table 2). Support for these birds’ being regarded as vectors of *V*. *cholerae* is the fact that the bacterium was isolated from a wild cormorant, and the genus *Vibrio* was identified in five out of seven wild birds by Illumina sequencing. *ompW* gene was detected in five wild cormorants, which supports the assumption that these *Vibrios* OTUs are in fact *V*. *cholerae* (Fig. 5). A very interesting result was the direct detection of *ctxA* in three wild cormorants, demonstrating that the birds harbored pathogenic strains of *V*. *cholerae*.

On migrating, great cormorants can cover distances of 500 to 1,000 km/day^37^, and thus potentially transfer *V*. *cholerae* across and between continents. Assuming that different bird species may be infected with different pandemic *V*. *cholerae* strains, and that their transmittance occurs under natural conditions, we conclude that birds act as vectors aiding the spread of the disease. Our findings further explain two important aspects: the long-distance transmittance of cholera and the speed at which it spreads.

As far as we know, our study is the first to investigate cormorant intestine microbiota. The rarefaction curves (Fig. S1) did not reach a plateau, suggesting that the actual bacterial diversity is much higher. The Venn diagram (Fig. 3) revealed that only 129 OTUs were found in all three intestinal sections. These results can be explained by the fact that each intestine section has its own unique role in food digestion, and its diverse environmental conditions such as oxygen concentrations, pH, etc., which may lead to the adaptation of a specific bacterial community. The microbiome analyses demonstrated that each individual cormorant possessed its own distinct microbiome (Fig. 4). The uniqueness of each individual can be explained by several factors such as the bird’s age, health status, different food consumption/dietary specialization, different environments, etc. Birds ringing (a numbered tag attachment to the leg or wing) enable birds’ individual identification and provide information regarding their life history including age, migration routes, feeding behavior and more. However, as rings were not detected on the wild cormorants that we sampled, these parameters were not available in the current study. Several studies have demonstrated wide variations in overall gut microbiome in individuals of different populations and from different environments^38–40^.

Diets can be a major non-genetic factor that governs gut microbiome. The effect of diets on the gut microbiota composition can be explained by two hypotheses; (i) each food item carries a unique bacterial assemblage; (ii) each food item supplies unique chemical compounds that support different bacterial assemblages. Experimental manipulation of avian diets has been shown to alter the gut microbiome within individuals over time, lending support to these hypotheses^41–45^.

The dominant phyla of the wild cormorants were *Fusobacteria*, *Firmicutes* and *Proteobacteria* (Fig. 2a and b). *Actinobacteria* and *Bacteroidetes* were present but much less abundant. Similar results were obtained for the intestinal microbiota of migrating shorebirds and artificial breeding geese^46, 47^. Wild bar-headed goose (*Anser indicus*)^47^ presented a different microbiome, with *Firmicutes*, *Proteobacteria*, *Actinobacteria* and *Bacteroidetes* as the dominant phyla. *Fusobacteria* was not detected at all in the gut microbiome of terrestrial avian, and *Bacteroidetes* and *Actinobateria* were much more abundant than they were in the cormorants in the current study or in the other waterbirds discussed above^48–50^.

Species of the *Fusobacteria* phylum are obligate anaerobic Gram-negative rods^51^. Three main *Fusobacteria* OTUs were found in the cormorants: *Fusobacterium* (39.3%), *Cetobacterium* (9.6%) and an unclassified OTU (50.9%) (Fig. 1). *Fusobacterium* and *Cetobacterium* species were found to inhabit intestinal tracts of humans and animals^52, 53^. Interestingly, these were identified as the main genera in class *Fusobacteria* in fecal samples of three shorebird species^46^.

Some bacterial genera identified in the cormorants’ intestine are potentially human or bird pathogens (Table S2). Cormorants may disseminate these pathogens globally, as was discussed here for *V*. *cholerae* and in the literature^6, 9, 46, 54^. For example: *Corynebacterium*, *Campylobacter*, *Helicobacter* and *Clostridium* were detected in all the birds’ intestine samples at average frequencies of 0.35%, 4.73%, 0.91% and 22.16%, respectively. Ryu *et al*.^46^ also reported the presence of *Campylobacter*, *Helicobacter* and *Clostridium* in fecal samples of red knot, ruddy turnstone and semipalmated sandpiper, collected during a migratory stopover in Delaware Bay.

In the cormorants’ fish-feeding experiments we isolated 48 and 141 *V*. *cholerae* strains from fish and cormorants, respectively. These isolates were all non O1/non O139, positive for *toxR* genes and negative for *ctxA*, *tcpA*, and *tcpI*. The majority ( >90%) were positive for the *hapA* and *hylA* genes, and 30% possessed *ompU* and TTSS genes. *zot* gene was present in about 20% of the isolates (Table 3). All these genes are associated with the pathogenicity potential of *V*. *cholerae* ^55–57^. For example, the presence of *hlyA* gene was reported to cause symptoms similar to cholera toxin in hospitalized diarrheal patients^56, 58^.

### In conclusion

Cholera is a devastating disease that spreads globally, but the ways of its dissemination are as yet unknown. Here we showed that cormorants can become infected with *V*. *cholerae* by consuming fish infected with this bacterium. Moreover, *V*. *cholerae* was isolated from a wild cormorant and was molecularly detected in five. The gene for A subunit of cholera toxin (*ctxA*) was also detected in three of the wild birds’ intestine samples. The current study is the first evidence that waterbirds can be infected with *V*. *cholerae* from their fish prey. The large-scale movements of cormorants as well as many other fish-eating waterbirds provide a potential mechanism for the global distribution of *V*. *cholerae*. Further research should be performed in areas endemic to pathogenic strains of *V*. *cholerae* and with various bird and fish species.

## Methods

### Ethics Statement

All methods were performed in accordance with the relevant guidelines and regulations. Great cormorants are subject to controlled culling because of their alleged damage to fisheries^30^. Israel and all member states of the EU are party to the African-Eurasian Waterbird Agreement (AEWA) under the Convention on Migratory Species (CMS) of the United Nations Environmental Program (UNEP), (www.unep-aewa.org). Hunting permission for controlled waterbirds accords with regulations of wild animals protection 1955 and 1976, [regulation #5 A(2–4), 1976; in Hebrew]. Seven wild cormorants were collected after being shot by fishermen next to their fish ponds. In addition, Cormorants feces were collected from hand-reared birds (n = 8) kept in captivity in a zoological garden at Oranim, Tivon. All procedures were performed under the consent of the cormorants’ owners and under permits of the Israel Nature Parks Authority, University of Haifa Ethics Committee and the Israel Committee for Ethics in Animal Experimentation (permit #033-b4822-1-02/02/2012). All the fish in the current study were obtained at different locations from fishermen selling fresh fish for consumption.

### Sampling microbiota in wild great cormorant’s intestine

We examined the intestine of seven great cormorant birds that in Israel are subject to controlled culling because of their alleged damage to fisheries^30^. Six birds were from Ma’agan Michael (32°33'31.6''N 34°54'37.6''E), and one was from Beit She’an valley (32°29 05.4''N 35°31'45.9''E). They were collected immediately after being shot by fishermen beside their commercial fish pond and brought directly into the lab. Three sections of the intestine were sampled for the presence of *V*. *cholerae* and for the microbiome analyses: (A) close to the esophagus (hereinafter esophagus), (B) the middle of the intestine (hereinafter middle), and (C) the cloaca region (hereinafter cloaca). For the microbiome analyses 0.5 gr of the intestine content from each intestine part was transferred to a 2 ml sterile tube that contained 0.5 ml of absolute ethanol. Tubes were kept at −20 °C until DNA extraction. For *V*. *cholerae* detection, intestine samples were treated as described below.

### DNA extraction from intestine samples

DNA was extracted from the intestinal samples with a DNA isolation kit (DNeasy Blood and Tissue, Qiagene, Germany) according to the manufacturer’s instructions with minor modifications. To obtain DNA without the ethanol residues, the tubes were centrifuged for 30 min at maximum speed, and the ethanol was removed from the tube. Next, 180 µl ELB (Enzymatic lysis buffer- 20 mM Tris HCL pH 8, 2 mM sodium EDTA and 1.2% Triton-X-100) were added to the sample with 20 mg/ml lysozyme (from chicken egg white, SERVE, Germany) and the samples were incubated with shaking for 60 minutes at 37 °C. Extraction then continued according to the manufacturer’s instructions, with storage at −20 °C.

### Generation of the 16S rRNA gene library

A set of primers was used to amplify the V4 variable region of the 16S rRNA gene: CS1_515F (ACACTGACGACATGGTTCTACAGTGCCAGCMGCCGCGGTAA) CS2_806R (TACGGTAGCAGAGACTTGGTCTGGACTACHVGGGTWTCTAAT) (primers from: Sigma Aldrich, Israel) as described previously by Caporaso *et al*.^59^.

PCR amplification was performed using the EmeraldAmp MAX HS PCR Master Mix (Takara bio Inc, Otsu, Shiga, Japan) in a total reaction of 25 µl. The final concentration of each primer was 0.5 ng/µl. 10–100 ng genomic DNA were added to each PCR reaction. PCR conditions were 95 °C for 5 min, followed by 28 cycles of 30 sec at 95 °C; 45 sec at 55 °C; and 30 sec at 68 °C. The final step was 7 min at 68 °C. Reactions were verified to contain visible amplification using 1.5% agarose gel electrophoresis.

### Illumina MiSeq sequencing

Illumina MiSeq sequencing was performed at the DNA Services (DNAS) facility - University of Illinois at Chicago (UIC). The sequencing protocol is described in detail in Aizenberg-Gershtein *et al*.^60^ The procedure included a second PCR amplification with a separate primer pair for sample, obtained from the Access Array Barcode Library for Illumina, where each sample received a separate primer set with a unique 10-base barcode (Fluidigm, South San Francisco, CA; Item #100-4876). Pooled, diluted libraries were sequenced with Illumina MiSeq. 600-cycle sequencing kit version 3, and analyzed with Casava 1.8 (pipeline 1.8). Reads were 200 nucleotides in length (paired end, 2 × 200). PhiX DNA was used as a spike-in control. Barcode sequences from Fluidigm were provided to the MiSeq server, and sequences were automatically binned according to 10-base multiplex identifier (MID) sequences. Raw reads were recovered as FASTQ files.

### Sequence analysis

Bioinformatics analysis was performed on MOTHUR v.1.33.338. The operational taxonomic unit (OTU)-based approach of the MiSeq Standard Operating Procedure (SOP) was followed^61^. Any sequences with uncertainties or homopolymers longer than 8 bases were removed from the data set. Sequences were aligned using the SILVA-compatible alignment database available within MOTHUR. Sequences were trimmed to a uniform length of 295 base pairs, and chimeric sequences were removed with Uchime^62^. Sequences were classified using the MOTHUR-formatted version of the RDP training set (v.9), and clustered into OTUs based on 97% sequence identity; then the entire dataset was randomly subsampled to the minimum number of sequences per sample (lowest number of sequences obtained in a sample): 24,901 sequences per sample.

All the sequence data analyses reported in this paper can be downloaded from the National Center for Biotechnology Information NCBI (https://www.ncbi.nlm.nih.gov/sra) BioProject number PRJNA336254 (items 25–31).

### Microbial richness and similarity estimates of wild cormorants

#### Alpha Diversity

To evaluate the microbial richness of the cormorant’s intestine community, we calculated the estimated “true” species diversity using the chao1 estimator. This index was calculated for the different intestine sections and for each individual bird (when all the sections were pooled), using the subsampled OTUs and genera tables. Chao1 is a nonparametric, abundance-based richness estimator, and was calculated using the observed number of OTUs (sobs) and the fraction between the number of the singletons and doubletons^63^. This index was calculated using the software EstimateS (Version 9.1.0).

#### β-diversity

To predict the β-diversity in the different birds and intestine sections (esophagus, middle, cloaca), we processed the data by the MOTHUR program (version 1.33.3) as described previously by Kozich *et al*.^61^. The following statistic parameters were calculated: (1) A Venn diagram was created to show the overlapping and unique OTUs found in each intestine part. (2) Ordination plots using the thetaYC distance matrix among samples were plotted to create a three-dimensional non-metric multidimensional scaling (nMDS). Additionally, Analysis of Similarities (ANOSIM) was conducted to test each of the studied wild cormorants (n = 7) for differences in their bacterial communities’ composition. ANOSIM (“R” software with “vegan” package) was applied using OTUs’ relative abundances with Bray-Curtis distance index (permutations = 1000).

### Direct detection of *V. cholerae* from DNA intestine samples

To verify the presence of *V. cholerae* in the wild cormorant’s intestine we performed a multiplex PCR in accordance with Nandi *et al*.^64.]^ This PCR identifies the presence of an outer membrane protein gene *ompW*, which is specific for *V*. *cholerae*, and the *ctxA* gene (A subunit of cholera toxin).

### Isolation and Identification of *V. cholerae*

The presence of culturable *V*. *cholerae* was examined in the intestine content samples from the seven wild cormorants (described above), the cormorants’ feces samples (feeding experiments) and from fish intestine samples (fish from the feeding experiments). Thiosulfate-citrate-bile salts-sucrose (TCBS) agar plates (Difco, USA) were used for isolation and cultivation of *V*. *cholerae*. Intestine or feces samples were spread directly on the TCBS plates without any enrichment for *V*. *cholerae*. TCBS plates were incubated at 37 °C for 24 h and yellow colonies suspected as being *V*. *cholerae* were sub-cultured five times on LB agar plates for colonies’ isolation. Isolates’ identity was verified by multiplex PCR assay according to Nandi *et al*.^64^. After *V*. *cholerae* identification, the isolates were further examined to determine whether they were members of the O1 and O139 serogroups, by slide agglutination with use of two specific antisera: (1) a poly antiserum specific for O1 surface antigen (Difco), and (2) an antiserum specific for O139 surface antigen (Ministry of Health, Israel). Identified isolates were kept in LB with 30% glycerol (Hilabs, Mumbai) at −80 °C.

### Cormorants’ feeding experiment

To determine if *V*. *cholerae* content in the cormorants’ feces changed with feeding regime, we tested hand-reared birds (n = 8) kept in captivity in a zoological garden at Oranim, Tivon. Prior to the feeding experiments, the cormorants were fed on goldfish (*Carassius auratus*) or koi (*Cyprinos carpio*). The fish were kept frozen at −20 °C for at least 30 days before they were used for the feeding experiment. They were thawed before being given to the birds. We screened the intestines of goldfish (n = 10) and koi (n = 10) for culturable *V*. *cholerae* according to the protocol described above, and all samples were found negative. Later we screened the feces of all the cormorants and all samples were found *V*. *cholerae*-negative. By contrast, the intestines of fresh collected tilapia (*Oreochromis niloticus* X *Oreochromis aureus*; n = 10), or tilapia frozen for up to 10 days (at −20 °C), were found positive for *V*. *cholerae*.

For the experimental procedure, the cormorants were divided into two separate groups (A and B) and fed on tilapia or goldfish and koi, respectively. The feeding of each group on a given diet lasted two to three weeks, with the cormorants fed four times/week. The presence of *V*. *cholerae* in the intestine of the fish (n = 6) during the experimental period was determined weekly. After about three weeks of experiment, the diet of all the cormorants in both groups was switched to goldfish or koi, which were negative for *V*. *cholerae*. This interval between experiments lasted up to three weeks. During the interval, the cages and the water were cleaned and cormorant’s feces were checked for the presence of *V*. *cholerae*. The next experiment started with diet switching between the groups (A and B: see above) and only after verification that the water and the feces samples were all negative for *V*. *cholerae*. The experiments, including the intervals between them, were repeated seven times.

Feces were collected immediately after feeding, as well as occasionally on days when the birds were not fed and during the interval between the experiments. Feces were collected by means of bacteriological sterile needles. Samples were spread directly onto TCBS agar plates which were incubated for 24 h at 37 °C. In parallel, 100 ml water from the pools of both groups was filtered through a 0.2 µm filter (Sartorius, Germany) and filters were placed on TCBS medium for *V*. *cholerae* detection. All isolates were treated as described above.

### Detection of *V. cholerae* virulence genes

In addition to *ctxA*, the presence of other virulence genes was determined in all *V*. *cholerae* isolates. The genes were: zonula occludens toxin (*zot*), El Tor-like hemolysin (*hlyA*), haemagglutinin/protease (*hapA*), outer membrane protein (*ompU*), TCP expression (*tcpI* and *tcpA*) and the central regulatory protein (*toxR*). PCR procedures and primers are described in Halpern *et al*.^5^ and Senderovich *et al*.^13^. To test the TTSS cluster in this study we used the genes *vcsC2*, *vcsN2*, *vspD* and *vcsV2*, as described in Chatterjee *et al*.^56^ and in Dziejman *et al*.^55^.

## Electronic supplementary material

Supplementary file
