# Supplementary material for: Great cormorants (Phalacrocorax carbo) as potential vectors for the dispersal of Vibrio cholerae
Source: Sci Rep. 2017 Aug 11;7:7973. doi: 10.1038/s41598-017-08434-8 (PMC5554209; doi:10.1038/s41598-017-08434-8)
Supplement: Supplementary file 1 — Supplementary file [file 41598_2017_8434_MOESM1_ESM.pdf]

Great cormorants (*Phalacrocorax carbo*) as potential vectors for the dispersal of *Vibrio cholerae*

Sivan Laviad-Shitrit, Tidhar Lev-Ari, Gadi Katzir, Yehonatan Sharaby, Ido Izhaki and Malka Halpern

Supplementary Figures

Figure S1. **Rarefaction curves** indicating the observed number of operational taxonomic units (OTUs) at a phylogenetic distance of 3% sequence similarity in the intestine samples. Rarefaction curves present the increase in the number of the OTUs as a function of the sequences number of each individual sample.

Figure S2. Hand-reared cormorants feeding experiment. Detailed results for three experimental repetitions (birds fed on tilapia). **a,** experimental repetition no. 2; **b**, experimental repetition no. 6; **c**, experimental repetition no. 7.

Supplementary Tables

Table S1. Microbial richness of the seven cormorants (the three sampled intestine parts were combined for each bird) subsampled OTUs at the genera levels.

Table S2. Potential pathogenic genera that were identified in great wild cormorant intestines.

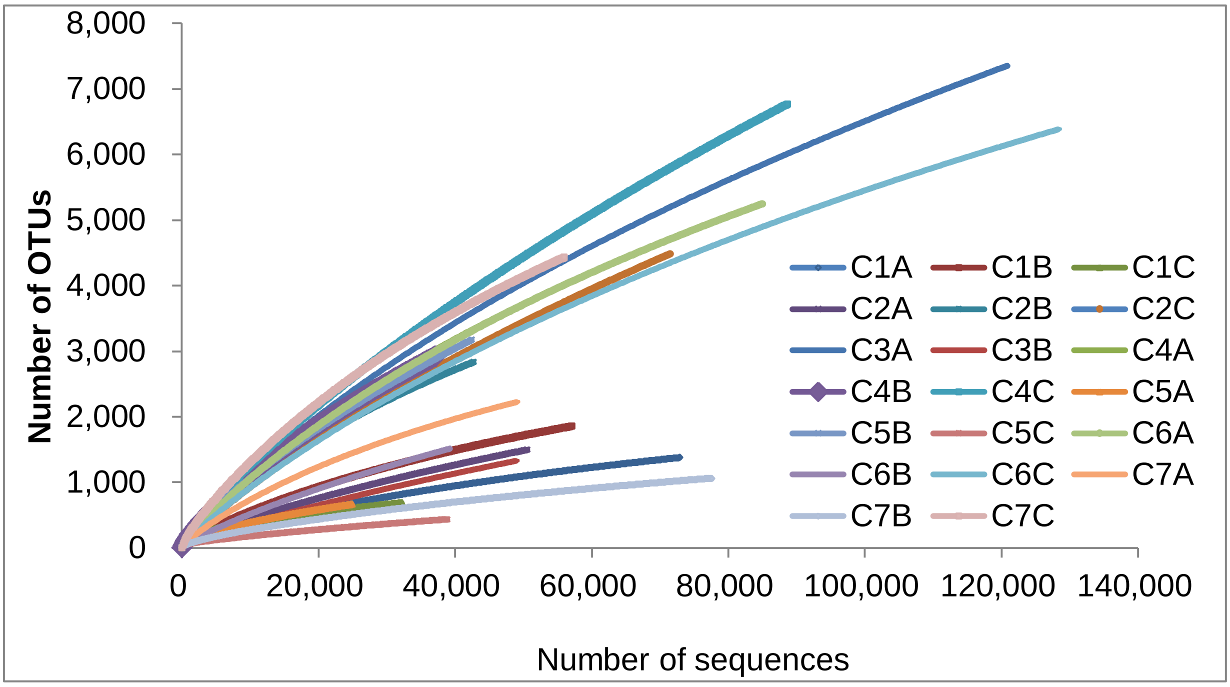

Figure S1. **Rarefaction curves** indicating the observed number of operational taxonomic units (OTUs) at a phylogenetic distance of 3% sequence similarity in the intestine samples. Rarefaction curves present the increase in the number of the OTUs as a function of the sequences number of each individual sample.

The explanation of the different samples name is as follows; The first letter C, indicates that the samples are from cormorants; the numbers indicate the cormorant individuals (from 1 to 7); the letters A, B, C at the end of the names indicate the three intestine parts; A - esophagus, B - middle, C - cloaca region.

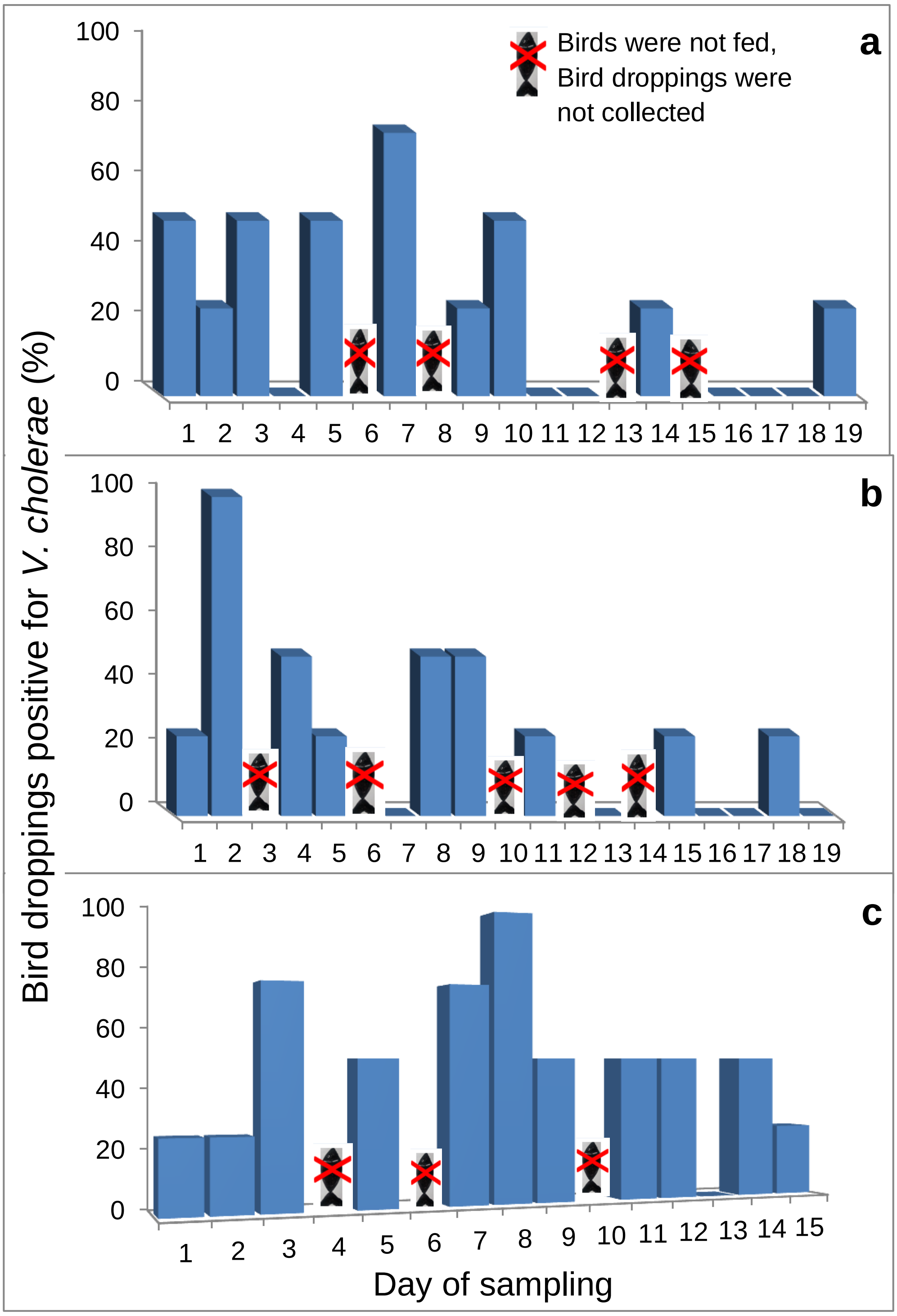

Figure S2. Hand-reared cormorants feeding experiment. Detailed results for three experimental repetitions (birds fed on tilapia). **a,** experimental repetition no. 2; **b**, experimental repetition no. 6; **c**, experimental repetition no. 7. Diet switching was repeated seven times. In the interval between experiments cormorants were fed exclusively on goldfish and koi for at least two weeks and until *V. cholerae* was not detected for at lease 10 days. On day 1, bird droppings were positive for *V. cholerae* about one-two hours after the birds were fed with tilapia. Fish marked with X represent days that the birds were not fed and droppings were not collected. (n=4)

Table S1. Microbial richness of the seven cormorants (the three sampled intestine parts were combined for each bird) subsampled OTUs at the genera levels. The indexes Sobs Mean and Chao1 were calculated on the EstimateS software (Version 9.1.0). Sobs Mean calculates the average number of all the taxonomic units in all the samples and Chao1 calculates the expected taxonomic richness for the complete collection of each area (see more details in the Methods section). C1-C7 indicates the different cormorant individuals.

**Phylogenetic level**		**C1**	**C2**	**C3**	**C4**	**C5**	**C6**	**C7**
OTU's	Sobs Mean±SD	178±54	244±78	192±99	377±15	206±134	220±74	260±167
	Chao1±SD	577±270	1739±934	1204±797	1809±176	834±912	1601±709	1294±949
All Genera	Sobs Mean±SD	187±9	122±6	121±6	142±7	153±7	140±7	117±7
	Chao1±SD	198±7	139±9	138±11	169±13	164±8	162±10	127±6

Table S2. Potential pathogenic genera that were identified in great wild cormorant intestines.

Genus	Positive birds (n)	Intestine sections	Symptoms and reference for humans	Symptoms and reference for birds
Actinobacteria				
Actinomyces	7	A-C	Cervicofacial, thoracic and abdominal dieses, infections of the female genital organs, the eye, the tissue adjacent to dental implantation elements, tooth extraction wounds[65]	-
Corynebacterium	7	A-C	Granulomatous lymphadenitis, pneumonitis, pharyngitis, cutaneous infections, endocarditis[66]	-
Rothia	5	A-C	Carious teeth, bronchial aspirate, postoperative wound[67]	-
Mycobacterium	5	A-C	Tuberculosis, leprosy[68]	Tuberculosis, parasitic diseases, mycobacteriosis[62,69-72]
Bacteroidetes				
Porphyromonas	4	A-C	Periodontitis[73]	-
Flavobacterium	4	A,C	Pneumonia , meningitis, bacteremia[74]	Pericarditis in chicken, tarsal, eye disease[75]
Fusobacteria				
Fusobacterium	7	A-C	Lemierre's syndrome, liver abscess, lung abscess, infections of female genital tract, skin infections[76]	-
Proteobacteria				
Delftia	3	A, B	Catheter-related infection[77]	-
Arcobacter	4	B, C	Watery diarrhea, abdominal pain, nausea, vomiting, fever[78]	-
Campylobacter	7	A-C	Gastrointestinal infection[79]	Infectious hepatitis in chickens[80]
Helicobacter	7	A-C	Gastric ulcer, lymphoma, gastric cancer[81]	-
Edwardsiella	3	A-C	Gastroenteritis, septicemia, bacteremia[82]	-
Escherichia/ Shigella	6	A-C	Gastroenteritis infections, urinary tract infections[83]	Extra-intestinal diseases in chickens, turkeys, other avian species[84,85]
Plesiomonas	6	A-C	Bacteremia, meningitis, acute gastroenteritis, diarrheal disease[86]	-

Yersinia	4	A-C	Plague disease and infections of the mesenteric lymph nodes[87]	Mortality[88]
Vibrio	5	A-C	Cholera, gastroenteritis, wound infections, septicemia[89]	-
Aeromonas	6	A-C	Intestinal and extra-intestinal illnesses[90]	Septicaemia, salpingitis, diarrhea, conjunctivitis, weight loss[91]
Halomonas	7	A-C	Infections and contamination in a dialysis center[92]	-
Haemophilus	5	A-C	Respiratory tract infection in adults, acute otitis media in children[93]	Influenza viruses[94]
Acinetobacter	5	A-C	Peritonitis, pneumonia and infections in the central nervous system, skin, soft tissue and bone[95,96]	-
Psychrobacter	1	C	Endocarditis and peritonitis[97]	-
Pseudomonas	7	A-C	Eye and skin diseases[98]	Rhinitis, sinusitis, laryngitis, septicaemia, haemorrhagic[99,100]
Stenotrophomonas	3	A-C	Nosocomial infections, bacteraemia, infections of the respiratory tracts and the urinary tracts, post-operative infections, ocular infections and a variety of other disease syndromes[101]	-
Enterococcus	2	A,C	Endocarditis, urinary tract infection, bacteremia, diverticulitis, meningitis[102]	Septicemic disease[103]
Firmicutes				
Bacillus	1	C	Anthrax , wound and burn infections, meningitis, respiratory and urinary infections[104]	-
Streptococcus	7	A-C	Meningitis, septicemia, endocarditis, arthritis, pneumonia[105, 106]	Septicemic disease[107]
Staphylococcus	6	A-C	Bacteraemia, respiratory and urinary tracts infections[108]	Vascular congestion and inflammation of internal organs[100]
Clostridium	7	A-C	Botulism, diarrhea[109]	Neuroparalytic disease, avian botulism[54,72,100,110,111]

References

65. Schaal, K. P. & Lee, H. J. *Actinomycete* infections in humans—a review. *Gene* 27. Pulverer G. Problems of human actinomycosis. *Postepy. Hig. Med. Dosw.* **115**, 201–211 (1992).

66. Lipsky, B. A., Goldberger, A. C., Tompkins, L. S. & Plorde, J. J. Infections caused by non diphtheria corynebacteria. *Rev. Infect. Dis.* **4**, 1220-1235 (1982).

67. Brown, J. M., Georg, L. K. & Waters, L. C. Laboratory identification of *Rothia dentocariosa* and its occurrence in human clinical materials. *Appl. Microbiol.* **17**, 150-156 (1969).

68. Cosma, C. L., Sherman, D. R. & Ramakrishnan, L. The secret lives of pathogenic *mycobacteria*. *Annu. Rev. Microbiol*. **57**, 641–676 (2003).

69. Davis, J. W., Anderson, R. C., Karstad, L. & Trainer, D. O. *Infectious and parasitic diseases of wild birds*. Iowa State University Press, Ames, Iowa. 344 (1971).

70. Smit, T., Eger, A., Haagsma, J. & Bakhuizen, T. Avian tuberculosis in wild birds in the Netherlands. *J. Wildlife Dis*. **23**, 485–487 (1987).

71. Hejlic, K. & Treml, F. The occurrence of avian mycobacteriosis in free-living birds at different epizootiological situations of poultry tuberculosis. *Vet. Med-Czech*. **38**, 305–317 (1993). [In Czech.]

72. Wobeser, G. A. Diseases of wild waterfowl, 2nd Edition. Plenum Press, New York. 324 (1997).

73. Yilmaz, O. The chronicles of *Porphyromonas gingivalis*: The microbium, the human oral epithelium and their interplay. *Microbiology*. **154**, 2897–2903 (2008).

74. Hsueh, P. R. *et al.* Flavobacterium indologenes bacteremia: Clinical and microbiological characteristics. *Clin. Infect. Dis*. **23**, 550–555 (1996).

75. Vancanneyt, M. *et al. Flavobacterium meningosepticum*, a pathogen in birds. *J. Clin. Microbiol*. **32**, 2398-2403 (1994).

76. Citron, D. M. Update on the taxonomy and clinical aspects of the genus *fusobacterium*. *Clin. Infect. Dis*. **35**, 22–27 (2002).

77. Preiswerk, B. *et al*. Human infection with *Delftia tsuruhatensis* isolated from a central venous catheter. *J. Med. Microbiol.* **60**, 246–248 (2011).

78. Vandenberg, O. *et al. Arcobacter* species in humans. *Emerg. Infect. Dis*. **10**, 1863–1867 (2004).

79. Romero, S., Archer, J. R., Hamacher, M. E., Bologna, S. M. & Schell, R. F. Case report of an unclassified microaerophilic bacterium associated with gastroenteritis. *J. Clin. Microbiol.* **26**, 142-143 (1988).

80. Smibert, R. M. Genus *Campylobacter*. In R. E. Buchanan and N. E. Gibbons (Eds). *Bergey's manual of determinative bacteriology*, 8th ed. The Williams & Wilkins Co., Baltimore. 207–212 (1974).

81. Ertem, D. Clinical practice: *Helicobacter pylori* infection in childhood. *Eur. J. Pediatr.* **172**, 1427–1434 (2013).

82. Janda, J. M. *et al.* Pathogenic properties of Edwardsiella species. *J. Clin. Microbiol.* **29**, 1997–2001 (1991).

83. Croxen, M. A. & Finlay, B. B. Molecular mechanisms of *Escherichia coli* pathogenicity. *Nat. Rev. Microbiol*. **8**, 26 –38 (2010).

84. Foster, G., Ross, H. M., Pennycott, T. W., Hopkins, G. F. & Mclaren, I. M. Isolation of *Escherichia coli* 186:K61 producing cyto-lethal distending toxin from wild birds of the finch family. *Lett. Appl. Microbiol*. **26**, 395–398 (1998).

85. Dho-Moulin, M. & Fairbrother, J. M. Avian pathogenic *Escherichia coli* (APEC). *Vet. Res.* **30**, 299–316 (1999).

86. Brenden, R. A., Miller, M. A. & Janda, J. M. Clinical disease spectrum and pathogenic factors associated with *Plesiomonas shigelloides* infections in humans. *Rev. Infect. Dis.* **10**, 303–16 (1988).

87. Pujol, C. & Bliska, J. B. Turning *Yersinia* pathogenesis outside in: Subversion of macrophage function by intracellular yersiniae. *Clin. Immunol*. **114**, 216–226 (2005).

88. Hamasaki, S. I., Hayashidani, H., Kaneko, K. I., Ogawa, M. & Shigeta, Y. A survey for *Yersinia pseudotuberculosis* in migratory birds in coastal Japan. *J. Wildlife Dis.* **25**, 401–403 (1989).

89. Daniels, N. S. & Shafaie, A. A review of pathogenic *Vibrio* infections for clinicals. *Infect. Med*. **217**, 665 – 685 (2000).

90. Laviad, S. & Halpern, M. Chironomids' relationship with *Aeromonas* species. *Front. Microbiol.* **7**, 736 (2016). doi.org/10.3389/fmicb.2016.00736

91. Awaad, M. H., Hatem, M. E., Wafaa, A., Asia, E. & Fathi, A. Certain epidemiological aspects of *Aeromonas hydrophila* infection in chickens. *J. Am. Sci*. **7**, 761-770 (2011).

92. Stevens, D. A., Hamilton, J. R., Johnson, N., Kim, K. K. & Lee, J. S. *Halomonas*, a newly recognized human pathogen causing infections and contamination in a dialysis center: Three new species. *Medicine* (Baltimore). **88**, 244–249 (2009).

93. Murphy, T. F. & Apicella, M. A. Nontypable *Haemophilus influenzae*: A review of clinical aspects, surface antigens, and the human immune response to infection. *Rev. Infect. Dis*. **9**, 1-15 (1987).

94. Kishida, N., Sakoda, Y., Eto, M., Sunaga, Y. & Kida, H. Co-infection of *Staphylococcus aureus* or *Haemophilus paragallinarum* exacerbates H9N2 influenza A virus infection in chickens. *Arch. Virol*. **149**, 2095–2104 (2004).

95. Lye, W. C., Lee, E. J. & Ang, K. K. Acinetobacter peritonitis in patients on CAPD: Characteristics and outcome. *Adv. Perit. Dial*. **7**, 176–179 (1991).

96. Peleg, A. Y., Seifert, H. & Paterson, D. L. *Acinetobacter baumannii*: Emergence of a successful pathogen. *Clin. Microbiol. Rev*. **21**, 538e58 (2008).

97. Gini, G. A. Ocular infection caused by *Psychrobacter immobilis* acquired in the hospital. *J. Clin. Microbiol*. **28**, 400- 401 (1990).

98. Mena, K. D. & Gerba, C. P. Risk assessment of *Pseudomonas aeruginosa* in water. *Rev. Environ. Contam. Toxicol.* **201**, 71-115 (2009).

99. Walkers, S. E., Sander, J. E., Cline, J. L. & Helton, J. S. Characterization of *Pseudomonas aeruginosa* isolates associated with mortality in broiler chicks. *Avian Dis.* **46**, 1045–1050 (2002).

100. Benskin, C. M. H., Wilson, K., Jones, K. & Hartley, I. R. Bacterial pathogens in wild birds: A review of frequency and effects of infection. *Biol. Rev.* **84**, 349–373 (2009).

101. Hauben, L., Vauterin, L., Moore, E. R. B., Hoste, M. & Swings, J. Genomic diversity of the genus *Stenotrophomonas*. *Int. J. Syst. Bacteriol.* **49**, 1749–1760 (1999).

102. Morrison, D., Woodford, N. & Cookson, B. Enterococci as emerging pathogens of humans. *Soc. Appl. Bacteriol. Symp. Ser.* **26**, 89S–99S (1997).

103. Devriese, L. A. *et al. Enterococcus hirae* infections in psittacine birds: Epidemiological, pathological and bacteriological observations. *Avian Pathol.* **24**, 523–531 (1995).

104. Turnbull, P. C. B. & Kramer, J. M. *Bacillus*. In Murray, P. R., Baron, E. J., Pfaller, M. A., Tenover, F. C. & Yolken, R. H. (Eds.). *Manual of clinical microbiology* (American Society for Microbiology, Washington, D.C.) 6th ed. 349–356 (1995).

105. Lun, Z. R., Wang, Q. P., Chen, X. G., Li, A. X. & Zhu, X. Q. *Streptococcus suis*: An emerging zoonotic pathogen. *Lancet Infect. Dis.* **7**, 201–09 (2007).

106. Guiral, S., Mitchell, T. J., Martin, B. & Claverys, J. P. Competence-programmed predation of noncompetent cells in the human pathogen *Streptococcus pneumoniae*: Genetic requirements. *Proc. Natl. Acad. Sci. USA.* **102**, 8710-8715 (2005).

107. Droual, R., Ghazikhanian, G. Y., Shivaprasad, H. L., Barr, B. C. & Bland, M. B. *Streptococcus bovis* infection in turkey poults. *Avian Pathol.* **26**, 433–439 (1997).

108. Oliveira, D. C., Tomasz, A. & de Lencastre, H. Secrets of success of a human pathogen: Molecular evolution of pandemic clones of methicillin-resistant *Staphylococcus aureus*. *Lancet Infect. Dis.* **2**, 180–189 (2002).

109. Lyerly, D. M., Krivan, H. C. & Wilkins, T.D. *Clostridium difficile*: Its disease and toxins. *Clin. Microbiol. Rev.* **1**, 1-18 (1988).

110. Ankerberg, C. W. Pelican deaths in the vicinity of a sewage lift station: A bacteriological investigation. *Microbios. Letters.* **26**, 33–42 (1984).

111. Petermann, S., Glu¨nder, G., Heffels-Redmann, U. & Hinz, K. H. Untersuchungsbefunde an ‘‘krank’’ bzw. ‘‘tot’’ gefundenen Trottellummen (Uria aalge), Dreizehen- (Rissa tridactyla), Silber- (Larus argentatus) und Lachmo ̈wen (Larus ridibundus) aus dem Bereich der Deutschen Bucht, 1982–1985. Deutsche Tiera¨rztliche *Wochenschrift.* **96**, 271–277 (1989).
